# Overview on how oncogenic Kras promotes pancreatic carcinogenesis by inducing low intracellular ROS levels

**DOI:** 10.3389/fphys.2013.00246

**Published:** 2013-09-12

**Authors:** Bo Kong, Chengjia Qia, Mert Erkan, Jörg Kleeff, Christoph W. Michalski

**Affiliations:** ^1^Department of Surgery, Technische Universität MünchenMunich, Germany; ^2^Division of Surgical Oncology and Division of Abdominal Organ Transplantation, Department of Surgery, Oregon Health and Science UniversityPortland, OR, USA

**Keywords:** pancreatic cancer, redox equilibrium, reactive oxygen species, oncogenic Kras, pancreatic cancer stem cells, Kras^G12D^

## Abstract

Pancreatic ductal adenocarcinoma (PDAC) is a devastating disease without clearly known disease causes. Recent epidemiological and animal studies suggest that the supplementation of dietary antioxidants (e.g., vitamins C and E) decreases cancer risk, implying that increased reactive oxygen species (ROS) may play a role in pancreatic carcinogenesis. However, oncogenic Kras mutations (e.g., Kras^G12D^), which are present in more than 90% of PDAC, have been proven to foster low intracellular ROS levels. Here, oncogenic Kras activates expression of a series of anti-oxidant genes via Nrf2 (nuclear factor, erythroid derived 2, like 2) and also mediates an unusual metabolic pathway of glutamine to generate NADPH. This can then be used as the reducing power for ROS detoxification, leading collectively to low ROS levels in pancreatic pre-neoplastic cells and in cancer cells. In adult stem cells and cancer stem cells, low ROS levels have been associated with the formation of a proliferation-permissive intracellular environment and with perseverance of self-renewal capacities. Therefore, it is conceivable that low intracellular ROS levels may contribute significantly to oncogenic Kras-mediated PDAC formation.

## Introduction

Pancreatic ductal adenocarcinoma (PDAC) is a highly aggressive tumor entity without clearly known disease causes (Kong et al., 2011; Siegel et al., 2013). Oncogenic *KRAS* (v-Ki-ras2 Kirsten rat sarcoma viral oncogene homolog) mutations (e.g., *KRAS*^G12D^ or *KRAS*^G12V^) have been considered as the initiating genetic event for this disease (Kong et al., 2011). Recently, prospective studies have demonstrated that dietary antioxidants (e.g., vitamins C and E) significantly decreased cancer risk, underscoring an important role of the redox equilibrium in the etiology of PDAC (Gong et al., 2010; Banim et al., 2012; Heinen et al., 2012). Furthermore, genetic variations in antioxidant genes seem to modify the risk to develop PDAC in humans (Tang et al., 2010). In line, long-term treatment with δ-tocotrienol (a bioactive vitamin E derivative) which has putative anti-oxidative activity dramatically inhibited *Kras*^G12D^-driven formation of pancreatic intraepithelial neoplasms (mPanINs) in a genetically engineered mouse model (GEMM) of pancreatic cancer (Husain et al., 2011, 2013; Shin-Kang et al., 2011). These data suggest that a systemic reduction in the production of reactive oxygen species (ROS) may prevent/delay the development of PDAC. Paradoxically, recent studies have also demonstrated that oncogenic Kras^G12D^ mediates activation of metabolic programs, which effectively detoxify ROS and thus reduce ROS levels in pancreatic pre-neoplastic cells and in cancer cells. Furthermore, low intracellular ROS levels seem to be essential for Kras^G12D^-driven carcinogenesis in mice (deNicola et al., 2011; Son et al., 2013). In this case, a “chemo-” preventive effect of dietary antioxidants cannot be explained by reduced intracellular ROS levels in pancreatic epithelial cells. Thus, we reviewed and discussed the potential biological significance of Kras^G12D^-mediated ROS-detoxifying networks.

## Oncogenic kras initiates pancreatic cancer

Characterization of human cancer genomes confirmed that more than 90% of human PDACs harbor oncogenic KRAS mutations (Almoguera et al., 1988; Smit et al., 1988). The mutated *KRAS* encodes a protein locked in a constitutively active state, leading to persistent downstream signals such as activation of the RAF-MEK-ERK (extracellular signal-regulated kinase) cascade (Barbacid, 1987). The ability of oncogenic KRAS in initiating PDAC has been demonstrated in GEMMs of pancreatic cancer. Here, pancreas-specific expression of Kras^G12D^ recapitulated the whole spectrum of human PDAC pathologies, from its precursor lesions to locally invasive and metastatic entities (Hingorani et al., 2003). Recent studies have demonstrated that the activity of Kras^G12D^ is required for all stages of carcinogenesis including inception, progression and metastasis because inactivation of Kras^G12D^ using genetic approaches invariably reversed the ongoing carcinogenic process (Collins et al., 2012). However, it remains largely elusive how Kras^G12D^ exactly promotes PDAC development.

## Reactive oxygen species (ROS) metabolism

Chemically reactive molecules containing oxygen, which are usually termed as ROS, consist of free radical ROS [e.g., oxygen ions (O^−^_2_)] and non-radical ROS [e.g., peroxide (H_2_O_2_)]. The free radical ROS has unpaired electrons in the molecular orbital whereas non-radical ROS contains no unpaired electrons (Shi et al., 2012). ROS formation, as a natural byproduct of aerobic metabolism, can be derived from exogenous and endogenous sources (Castro and Freeman, 2001). As for the exogenous sources, substances (e.g., metals and chemicals) inducing ROS formation can be directly metabolized to radicals in cells or can trigger intracellular ROS production (Bonney et al., 1991; Halliwell and Aruoma, 1991; Dreher and Junod, 1996; Jaruga and Dizdaroglu, 1996; Wang et al., 1998). Under physiological circumstances, the mitochondrion is an intracellular organelle which is responsible for energy production through cellular respiration. However, the leaking electron from the mitochondrial electron transport chain eventually interacts with oxygen and generates superoxide radicals, producing approximately 98% of the endogenous ROS (Freeman and Crapo, 1982; McCord, 2000; Salvador et al., 2001). Apart from the mitochondrion, biochemical reactions within the endoplasmatic reticulum (ER), the peroxisome or the cytoplasm also generate additional ROS (Butler and Hoey, 1993; Conner and Grisham, 1996; Li and Jackson, 2002; Klaunig and Kamendulis, 2004; Valko et al., 2004). For instance, cytochrome P450 in the ER uses oxygen to oxidize and to detoxify foreign compounds; a process in which ROS are generated (Butler and Hoey, 1993). In addition, membrane-bound NADPH (nicotinamide adenine dinucleotide phosphate) oxidase in immune cells (e.g., neutrophils and macrophages) produces ROS via a biochemical process known as the respiratory burst, which is essential for these cells to eliminate bacteria (Conner and Grisham, 1996).

Since excessive ROS can cause oxidative damage to macromolecules (e.g., DNA and lipids) and can alter intracellular signal transduction (e.g., through NF-κB), intracellular ROS is constantly eliminated via a sophisticated ROS-detoxifying system including non-enzymatic antioxidants (e.g., Vitamins C and E) and enzymatic antioxidants [such as superoxide dismutase (SOD), catalase (CAT) and glutathione peroxides (GPX)] (Mates et al., 1999; McCall and Frei, 1999). Notably, the majority of these enzymes require the activity of reduced glutathione (GSH) which further relies on NADPH. In this case, NADPH provides the ultimate reducing power for ROS detoxification. Taken together, both non-enzymatic antioxidants and enzymatic antioxidants act as an “antioxidant network” which maintains a fine-tuned intracellular redox balance (Sies et al., 2005).

## Kras^G12D^ maintains low ROS levels in PDAC cells

**Figure 1:**
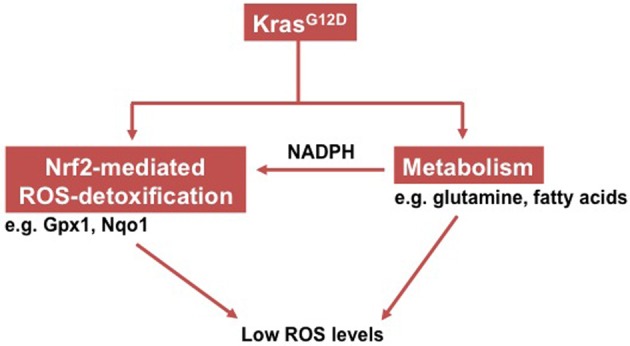
**Schema showing how oncogenic Kras induces low intracellular ROS levels**.

Until today, it remains elusive how Kras^G12D^ promotes PDAC. Recent studies demonstrated that Kras^G12D^ induces maintenance of low intracellular ROS levels via the transcription factor Nrf2 (nuclear factor, erythroid derived 2, like 2), which is a master switch in the antioxidant network (deNicola et al., 2011). To provide a reducing power for this Nrf2-mediated antioxidant program, Kras^G12D^ promotes a concerted metabolic program (e.g., thorough glutamine and fatty acid) that continually sustains the intracellular NADPH/NADP^+^ ratio (Khasawneh et al., 2009; Son et al., 2013) (Figure 1).

An earlier study demonstrated that ectopic expression of oncogenic Ras increased ROS production through NADPH-oxidase (Nox; Irani et al., 1997). Later on, a follow-up study provided evidence that such an increased ROS generation is functionally relevant to oncogenic Ras-mediated malignant transformation of NIH3T3 cells (Mitsushita et al., 2004). However, this concept has been challenged by a recent study which substantiated that ROS production was actually repressed by endogenous expression of the Kras^G12D^ allele in mouse cell lines (deNicola et al., 2011). Further investigation uncovered that Kras^G12D^ activated Nrf2 via MAPK pathways (mitogen-activated protein kinase), which then initiated a set of antioxidant programs. Consistently, human PanINs and PDACs exhibit activation of NRF2 and have low ROS levels in comparison to normal pancreatic ducts cells. NRF2, which is negatively regulated by KEAP1 (kelch-like ECH-associated protein 1), controls the expression of a series of proteins involved in different steps of ROS detoxification—such as NADPH generation (Cullinan et al., 2004; McMahon et al., 2006; Hayes and McMahon, 2009). Unlike many other tumor entities such as lung cancers (Shibata et al., 2008; Kim et al., 2010), however, PDACs rarely harbor somatic mutations in either the *KEAP1* or *NRF2* genes that usually result in an active NRF2. Hence, the Nrf2-mediated antioxidant program in PDAC is activated in an oncogenic Kras-dependent manner. In line, silencing of Kras or blockade of the MAPK pathway effectively decreased Nrf2 expression and increased intracellular ROS levels.

As early illustrated, ROS detoxification is a biochemical process that consumes NADPH (NADPH provides the reducing power). Thus, generation and maintenance of constant intracellular NADPH levels is essentially important. In this regard, a previous study demonstrated that Kras^G12D^ enhanced glycolysis of PDAC cells and that it directed glycolytic intermediates into the non-oxidative pentose phosphate pathway (PPP) whereas the NAPDH-producing oxidative arm of the PPP remained unaffected (Ying et al., 2012). These data suggest that PDAC cells might use other NADPH-producing metabolic pathways to maintain intracellular NADPH levels. Indeed, a recent study uncovered a distinct metabolic pathway of glutamine which is used by PDAC cells to generate NADPH (Son et al., 2013). Briefly, glutamine-derived aspartate (Asp) and α-ketoglutarate (α-KG) are converted into oxaloacetate (OAA) via aspartate transaminase (GOT1). The OAA is metabolized into malate by malate dehydrogenase (MDH1) and subsequently into pyruvate by malic enzyme (ME1). Conversion from malate to pyruvate then creates NADPH, which is important for maintaining the redox balance of PDACs because inactivation of any component of this metabolic pathway increased intracellular ROS levels and affected tumor growth (Cairns et al., 2011). Though the tumor environment of PDAC is usually depleted of glutamine, a recent study substantiated that PDAC cells containing oncogenic Kras showed an increased protein uptake by macropinocytosis. These internalized proteins are then metabolized into glutamine that is fueled into the NDAPH-producing process (Commisso et al., 2013). It has also been demonstrated that the Kras^G12D^-expressing pancreas exhibited increased fatty acid oxidation (Khasawneh et al., 2009). Since fatty acid oxidation is a NADPH-generating process (Jeon et al., 2012), it remains to be defined whether increased fatty acid oxidation also contributes to the maintenance of NADPH levels.

In conclusion, collaboration between Nrf2-mediated ROS detoxification and the NADPH-generating metabolic program collectively contributes to a “reduced” intracellular environment (e.g., low ROS levels). Since both of these depend on the activity of oncogenic Kras, it is conceivable that such an intracellular environment constitutes an important step in pancreatic carcinogenesis.

## The tumor-suppressing function of antioxidants does not contradict tumor-promoting effects of oncogenic kras-mediated low intracellular ROS levels

As earlier illustrated, prospective studies have suggested an association between dietary antioxidants and a decreased risk for developing pancreatic cancer (Gong et al., 2010; Banim et al., 2012; Heinen et al., 2012). Besides, certain antioxidants (especially δ-tocotrienol) have chemo-preventive effects in GEMMs of pancreatic cancer (Husain et al., 2011, 2013; Shin-Kang et al., 2011). Interestingly, these data rather point to a tumor-suppressing function of antioxidants in pancreatic cancer. However, the emergence of this evidence does not necessarily argue against the tumor-promoting functions of oncogenic Kras-mediated low intracellular ROS levels. Firstly, the tumor-suppressing function of antioxidants may be attributed to their effects on the immune system and especially T cell immunity. Recently, it has been shown that antitumor T cell immunity plays a crucial role in the early stages of pancreatic carcinogenesis (Bayne et al., 2012; Pylayeva-Gupta et al., 2012). In this regard, the dietary supplementation of antioxidants (e.g., vitamins E or C) has been proven to significantly enhance T cell immunity in humans (Burgess and Johansen, 1976; Meydani et al., 1997; Malmberg et al., 2002). Therefore, antioxidants may execute their tumor-suppressing functions by promoting antitumor immunity. Secondly, it remains largely unknown whether ROS levels in the pancreas (especially in epithelial cells) are actually affected by the intake of dietary antioxidant in humans. Thus, it is difficult to evaluate the contribution of their antioxidative effects to the development of pancreatic cancer. Lastly, some antioxidants display antitumor activities independent of their antioxidative effects. For instance, δ-tocotrienol, which has been used for chemo-prevention of pancreatic cancer in animal studies, contains an unsaturated isoprenoid side chain that has a unique antitumor property (Shin-Kang et al., 2011). Taken together, further studies are required to clarify how/why antioxidants execute their tumor-suppressor functions on oncogenic Kras-mediated low intracellular ROS levels in the pancreas.

## Low ROS levels in different biological systems

Although the biological significance of such an oncogenic Kras-mediated reductive intracellular environment remains unclear, this phenomenon has been widely described in other biological systems (Table 1). For example, when yeast cells are cultured under nutrient-limited conditions, they display a periodic metabolic cycle alternating between glycolysis and respiration. Their cell cycle is tightly restricted to the reductive phase of the metabolic cycle, which guarantees that DNA replication only occurs during glycolysis when the oxidative damage from respiration on the genome is minimal. Such a circadian rhythm that coordinates the metabolic and cell division cycles in situations where resources are limited, simply reflects an evolutionarily conserved means of preserving genome integrity (Chen et al., 2007). Silencing of a DNA checkpoint kinase abolishing such a rhythm allows DNA synthesis outside of the reductive phase but at the cost of increased spontaneous mutation rates. In adult stem cells, a similar nutrient-limited microenvironment (hypoxia) with low intracellular ROS levels also exists (Suda et al., 2011; Zhang and Sadek, 2013). Here, low ROS levels have been shown to be essential for maintaining the stem cell functions of hematopoietic stem cells (HSCs) in that the ROS^low^ cell population expressed high levels of stemness-associated molecules such as Notch1 and telomerase; it also had a higher self-renewal potential than the ROS^high^ population of cells (Jang and Sharkis, 2007). Similarly, mammary epithelial stem cells have low ROS levels (Diehn et al., 2009). Low ROS levels have been described in another type of “stem” cells—the “cancer stem” cells (CSCs) or “tumor-initiating” cells (TICs) (Shi et al., 2012). Historically, CSCs have been defined as a subset of cancer cells that are responsible for initiation, maintenance and metastasis of cancer (Lapidot et al., 1994). A seminal study demonstrated that human breast CSCs contained lower ROS levels than their non-tumorigenic progeny (Diehn et al., 2009). These low ROS levels rendered the CSCs highly resistant toward irradiation-induced DNA damage and cell death. Consistently, a recent study provided functional evidence that CSC-like properties in basal-like breast cancer are induced, when ROS production is inhibited by metabolic reprogramming of glucose metabolism [e.g., when more NADPH is generated, (Dong et al., 2013)]. Taken together, low ROS levels in other biological systems appear to be associated with stemness properties of cells in mammals or with a proliferation-permissive intracellular environment in low eukaryotic systems, both of which may contribute to oncogenic Kras-mediated carcinogenesis in the pancreas.

**Table 1:** **Cellular systems with low intracellular ROS levels**.

**References**	**Species/organ system**	**Condition**	**Biological significance**
Chen et al., 2007	Yeast	Nutrient-limited	Preserve integrity of the genome
Jang and Sharkis, 2007	Mouse/hematopoietic stem cells	Hypoxic	Reserve stem cell function
Diehn et al., 2009	Mouse/mammary epithelial stem cells	–	Maintain stemness
Diehn et al., 2009	Human/breast CSCs	Cancer microenvironment	Preserve tumor-initiating capacity and radio-resistance
Dong et al., 2013	Cell lines/basal-like breast cancer CSCs	Inhibit ROS production by metabolic reprogramming	Promote CSC-like properties

## Low ROS levels and pancreatic carcinogenesis

Because early expansion of pancreatic stem/progenitor cells accelerates Kras^G12D^-driven carcinogenesis in mice (Kong et al., 2011), Kras^G12D^-induced low intracellular ROS levels may facilitate expansion of pancreatic stem/progenitor cells by creating a proliferation-permissive intracellular environment. Furthermore, despite a questionable general compliance of PDAC to the CSCs concept, the heterogeneity of pancreatic cancer tissues indicates that a subset of pancreatic cancer cells may have low intracellular ROS levels in comparison to others.

## Conclusion

The exact contribution of pre-neoplastic and cancer cells with low ROS levels to PDAC initiation, progression and metastasis in humans remains to be defined. Certainly, such a subset of cancer cells may constitute a promising drug target for future therapies. Though the Nrf2-mediated network has been proposed as a potential drug target (Arlt et al., 2012), further studies on the contribution of (low) ROS levels to the aggressiveness of pancreatic cancer are warranted.

### Conflict of interest statement

The authors declare that the research was conducted in the absence of any commercial or financial relationships that could be construed as a potential conflict of interest.
